# A novel nomogram and risk classification system predicting the overall survival of patients with papillary renal cell carcinoma after nephrectomy: A population-based study

**DOI:** 10.3389/fpubh.2022.989566

**Published:** 2022-10-05

**Authors:** Yongtao Hu, Shun Xu, Qiao Qi, Xuhong Wang, Jialin Meng, Jun Zhou, Zongyao Hao, Qianjun Liang, Xingliang Feng, Chaozhao Liang

**Affiliations:** ^1^Department of Urology, The First Affiliated Hospital of Anhui Medical University, Hefei, China; ^2^Institute of Urology, Anhui Medical University, Hefei, China; ^3^Anhui Province Key Laboratory of Genitourinary Diseases, Anhui Medical University, Hefei, China; ^4^Department of Urology, Lu'an Hospital of Anhui Medical University, Lu'an People's Hospital of Anhui Province, Lu'an, China; ^5^Department of Urology, The Third Affiliated Hospital of Soochow University, Changzhou, China

**Keywords:** papillary renal cell carcinoma, nomogram, overall survival, prognosis, SEER

## Abstract

**Background:**

Papillary renal cell carcinoma (pRCC) is the largest histologic subtype of non-clear-cell RCC. To date, there is no reliable nomogram to predict the prognosis of patients with pRCC after nephrectomy. We aimed to first establish an effective nomogram to predict the overall survival (OS) of patients with pRCC after nephrectomy.

**Methods:**

A total of 3,528 eligible patients with pRCC after nephrectomy were identified from the Surveillance, Epidemiology, and End Results (SEER) database between 2010 and 2015. The patients were randomized into the training cohort (*n* = 2,472) and the validation cohort (*n* = 1,056) at a 7:3 ratio. In total, 122 real-world samples from our institute (titled the AHMU-pRCC cohort) were used as the external validation cohort. Univariate and subsequent multivariate Cox regression analyses were conducted to identify OS-related prognostic factors, which were further used to establish a prognostic nomogram for predicting 1-, 3-, and 5-year OS probabilities. The performance of the nomogram was evaluated by using the concordance index (C-index), receiver operating characteristic curve (ROC), calibration plot, and decision curve analysis (DCA).

**Results:**

Multivariate Cox analysis showed that age, race, marital status, TNM stage, tumor size, and surgery were significant OS-related prognostic factors. A prognostic model consisting of these clinical parameters was developed and virtualized by a nomogram. High C-index and area under the ROC curve (AUC) values of the nomogram at 1, 3, and 5 years were found in the training, validation, and AHMU-pRCC cohorts. The calibration plot and DCA also showed that the nomogram had a satisfactory clinical application value. A risk classification system was established to risk-stratify patients with pRCC.

**Conclusion:**

Based on a large cohort from the public SEER database, a reliable nomogram predicting the OS of patients with pRCC after nephrectomy was constructed, which could optimize the survival assessment and clinical treatment.

## Introduction

Kidney cancer is the 13th most common malignant tumor globally and ranks third among genitourinary tumors after prostate cancer and bladder cancer, accounting for 2–3% of all adult organically sourced malignancies (1). Papillary renal cell carcinoma (pRCC), an aggressive urological malignancy originating from the renal parenchymal urothelial system, represents the second most common histopathological type of RCC, accounting for 15% of kidney-derived malignancies (2). Based on global cancer statistics and epidemiological data on kidney cancer, it was estimated that in 2020, at least 65,000 new pRCC cases were diagnosed all over the world (3). It is worth mentioning that the incidence of kidney cancer has been increasing steadily in most countries, increasing by 2% annually over the last decade (4–6). Furthermore, 17% of patients with RCC have distant metastasis at the time of diagnosis due to nonspecific clinical symptoms in the early stages (7), and 25% of patients with localized primary renal carcinoma even develop metastatic lesions after radical nephrectomy (8). However, metastatic patients harboring pRCC exhibit unfavorable survival outcomes even with different treatments (9). Therefore, as an important subtype of the RCC family, pRCC has become a growing concern in clinical practice.

Clinical and biological studies have proven that RCC is not a single tumor but a general concept of various types of cancer occurring in the kidney. Different histopathological types have their corresponding clinical characteristics, genetic profiles, and biological behaviors, which can lead to different oncologic outcomes (4, 10–12). Large population-based retrospective cohort studies compared the difference in survival outcomes between pRCC and clear-cell RCC, and the results showed that patients with nonmetastatic pRCC were associated with lower cancer-specific mortality than patients with clear-cell RCC (13). Nevertheless, patients with pRCC presented a significantly high risk of lymph node metastasis, and the oncologic outcomes of patients with metastatic pRCC were generally more unfavorable than those of patients with metastatic clear-cell RCC (14, 15). These findings further suggest that there are different survival outcomes between pRCC and clear-cell RCC. Consequently, taking all histopathological types of RCC into account and evaluating the prognosis are not appropriate (16). In addition, surgery is a significant prognostic factor for RCC survival. For patients with localized RCC, partial nephrectomy and radical nephrectomy are the standard treatments recommended by the guidelines (5). However, currently, few prediction tools focus on pRCC only, and the effects of nephrectomy on the OS of pRCC do not receive enough attention. To accurately evaluate the prognosis of patients with pRCC after nephrectomy, it is necessary to develop a postoperative survival assessment model.

Disease prediction models based on medical public databases have been used to assist in clinical decision-making (17, 18). For the reasons mentioned before, we attempted to first establish and validate a prognostic nomogram to predict the OS of patients with pRCC after nephrectomy using a large cohort from the public SEER database. In addition, the nomogram was compared with the traditional AJCC staging to demonstrate its higher clinical practicability. With the guidance of this novel nomogram, the decision-making of individualized treatment for patients with pRCC will be optimized.

## Materials and methods

### Patient selection

Patients with pRCC were retrieved from the SEER 18 Registries Research Plus Data (2000–2018) by applying SEER^*^Stat software version 8.3.9 after acquiring permission to exploit data for academic research (reference number: 15425-Nov2021). Data screening was performed by using Excel 2019. To obtain the data on tumor size and the latest clinical stage and ensure a relatively long follow-up period, the time of diagnosis was limited to between 2010 and 2015. For external validation, we extracted patient data from the Department of Urology, First Affiliated Hospital of Anhui Medical University. Patients enrolled in this study were based on the following criteria: (1) patients diagnosed with histologically confirmed pRCC (Primary Site code C64.9; ICD-O-3 code 8260/3); (2) patients with only one primary tumor (pRCC); (3) those who received partial nephrectomy (Surg Prim Site code 30) or radical nephrectomy (Surg Prim Site code 50); and (4) those with complete records of relevant clinical parameters, including demographic data (age, sex, race, and marital status at diagnosis), clinicopathological data (pathological grade, TNM stage, and tumor size), and surgery. Moreover, patients with the Tx stage, Nx stage, Mx stage, and unknown survival time were excluded from the nomogram construction. The final enrolled patients with pRCC in the SEER database were grouped randomly at a 7:3 ratio to form the training and validation sets using the “caret” package in R software.

### Variable selection

The following variables were collected from the SEER database: demographic variables (age, sex, race, and marital status at diagnosis), clinicopathological variables (pathological grade, TNM stage, and tumor size), therapeutic variables (chemotherapy, radiation, and surgery), year of diagnosis, vital status, and survival time. The transformed 8th American Joint Committee on Cancer (AJCC) staging was used for subsequent nomogram development. Age and tumor size were categorized according to X-tile software to determine the optimal cutoff values (Supplementary Figure 1). All the included variables were further transformed into categorical variables. Age was divided into ≤61, 62–68, and ≥69 years. Race was categorized into black, white, and other. Marital status at diagnosis was divided into married and other (single, widowed, divorced, or separated). Tumor size was grouped into three classes: ≤48, 49–93, and ≥94 mm. According to the degree of differentiation, the pathological grade was divided into well (grade I), moderate (grade II), poor differentiation (grade III), and undifferentiation (grade IV). Surgery was classified as partial nephrectomy and radical nephrectomy. Chemotherapy and radiotherapy were categorized as “yes” or “no/unknown”. The primary endpoint of this study was OS, which referred to the period from the time of diagnosis to the time of death due to any cause.

### Statistical analysis

R software version 4.1.2 was used to complete the statistical computations used in the study. The best cutoff values of continuous variables were set by X-tile software version 3.6.1 (19). All the variables were transformed into categorical variables and expressed as frequencies and proportions. Univariate Cox regression analysis was used to preliminarily screen for potential prognostic factors in the training cohort. The variables with statistically significant differences were then evaluated by multivariate Cox regression analysis to determine the final factors, which were used to establish the prognostic nomogram for predicting OS probabilities (20, 21). KM plots were generated to draw survival curves in different subgroups of included factors, and intergroup differences were compared by log-rank tests. Nomogram accuracy was measured using the C-index and AUC (22). The larger the values of the C-index and AUC, the higher the effectiveness of the nomogram. Values > 0.7 for the C-index and the AUC were considered acceptable. Calibration plots with 1,000 bootstrap resampling were applied to compare the predicted survival with observed survival (23). The differences in clinical utility and benefit between the nomogram and the AJCC staging were compared by DCA (24). During the process of validation, each patient in the validation cohort was scored based on the nomogram constructed in the training cohort, and ROC, calibration plot, and DCA were drawn according to the final total points (25). *P* < 0.05 was considered statistically significant.

## Results

### Clinical features

A total of 76,227 patients with kidney cancer were collected in the public SEER database between 2010 and 2015. Based on the established inclusion criteria, 3,528 patients with pRCC after nephrectomy in the SEER database were included. After randomization, there were 2,472 patients with pRCC in the training cohort, and the remaining 1,056 patients were assigned to the internal validation cohort. A total of 122 eligible patients from the First Affiliated Hospital of Anhui Medical University were identified as the external validation cohort (AHMU-pRCC cohort). The demographic and clinicopathological data of eligible patients with pRCC from the SEER database are depicted in Table 1. The median age of the included patients was 61 (interquartile range, 53–68) years, including 2,571 male (72.9%) and 957 female patients (27.1%). Among all the patients, there were 1,038 blacks (29.4%), 2,329 whites (66.0%), and 161 of other races (4.6%). Married patients accounted for 62.0% of the cases, which was much larger than other patients. Moderate differentiation (51.4%) was the most common pathological grade, followed by poor differentiation (35.1%), well differentiation (10.0%), and undifferentiation (3.5%). According to the AJCC staging, there were 3,063 patients (86.8%) with T1/T2, 3,361 patients (95.3%) with N0, and 3,412 patients (96.7%) with M0. With regard to tumor size, there were 2,304 (23.5%), 878 (48.8%), and 346 patients (27.7%) with tumor sizes of ≤48 mm, 49–93 mm, and ≥94 mm, respectively. Regarding treatment information, nephrectomy was performed for all patients, while only a small proportion of patients were treated with radiation (0.8%) and chemotherapy (2.7%).

**Table 1:** Baseline characteristics of patients with papillary renal cell carcinoma after nephrectomy.

**Variables**	**All patients (*****n*** = **3,528)**		**Training cohort (*****n*** = **2,472)**		**Validation cohort (*****n*** = **1,056)**		***P*-value**
	***N***	**%**	***N***	**%**	***N***	**%**	
**Age**							0.386
≤61	1,856	52.6	1,318	53.3	538	50.9	
62–68	802	22.7	549	22.2	253	24.0	
≥69	870	24.7	605	24.5	265	25.1	
**Sex**							0.769
Male	2,571	72.9	1,805	73.0	766	72.5	
Female	957	27.1	667	27.0	290	27.5	
**Race**							0.448
Black	1,038	29.4	743	30.1	295	27.9	
White	2,329	66.0	1,617	65.4	712	67.4	
Other	161	4.6	112	4.5	49	4.6	
**Marital status**							0.313
Married	2,186	62.0	1,545	62.5	641	60.7	
Other	1,342	38.0	927	37.5	415	39.3	
**Year of diagnosis**							0.874
2010	511	14.5	355	14.4	156	14.8	
2011	560	15.9	401	16.2	159	15.1	
2012	549	15.6	382	15.5	167	15.8	
2013	585	16.6	411	16.6	174	16.5	
2014	629	17.8	447	18.1	182	17.2	
2015	694	19.7	476	19.3	218	20.6	
**Pathological grade**							0.541
I	352	10.0	240	9.7	112	10.6	
II	1,813	51.4	1,289	52.1	524	49.6	
III	1,239	35.1	859	34.7	380	36.0	
IV	124	3.5	84	3.4	40	3.8	
**T stage**							0.678
T1/T2	3,063	86.8	2,150	87.0	913	86.5	
T3/T4	465	13.2	322	13.0	143	13.5	
**N stage**							0.998
N0	3,361	95.3	2,355	95.3	1,006	95.3	
N1	167	4.7	117	4.7	50	4.7	
**M stage**							0.638
M0	3,412	96.7	2,393	96.8	1,019	96.5	
M1	116	3.3	79	3.2	37	3.5	
**Tumor size**							0.700
≤48	2,304	65.3	1,616	65.4	688	65.2	
49–93	878	24.9	608	24.6	270	25.6	
≥94	346	9.8	248	10.0	98	9.3	
**Chemotherapy**							0.117
Yes	97	2.7	61	2.5	36	3.4	
No/Unknown	3,431	97.3	2,411	97.5	1,020	96.6	
**Radiation**							0.418
Yes	27	0.8	17	0.7	10	0.9	
No/Unknown	3,501	99.2	2,455	99.3	1,046	99.1	
**Surgery**							0.604
Partial nephrectomy	1,941	55.0	1,353	54.7	588	55.7	
Radical nephrectomy	1,587	45.0	1,119	45.3	468	44.3	
**Vital status**							0.781
Alive	2,873	81.4	2,016	81.6	857	81.2	
Dead	655	18.6	456	18.4	199	18.8	

### Selection of prognostic factors

Most of the variables extracted from the SEER database were included in the analysis, except for the time of diagnosis, which was not suitable for nomogram development. Univariate Cox analysis identified three demographic variables (age, race, and marital status) and eight clinicopathological variables (e.g., pathological grade, TNM stage, tumor size, chemotherapy, radiation, and surgery) as significant candidate factors for OS in the training cohort (Table 2). These variables were further included in multivariate Cox analysis, and the final results showed that age, race, marital status, pathological grade, TNM stage, tumor size, and surgery were OS-related prognostic factors, while radiation and chemotherapy had no significant association with prognosis (Supplementary Figure 2).

**Table 2:** Univariate and multivariate Cox analyses of the training cohort on overall survival.

**Variables**	**Univariate analysis**			**Multivariate analysis**		
	**HR**	**95% CI**	***P*-value**	**HR**	**95% CI**	***P-*value**
**Age**						
≤61	Reference			Reference		
62–68	1.45	1.14–1.86	0.003*	1.56	1.21–2.00	<0.001*
≥69	2.56	2.08–3.16	<0.001*	2.67	2.15–3.32	<0.001*
**Sex**						
Male	Reference			–	–	–
Female	0.86	0.69–1.07	0.169	–	–	–
**Race**						
Black	Reference			Reference		
White	1.22	0.99–1.51	0.067	1.21	0.97–1.52	0.092
Other	2.34	1.60–3.41	<0.001*	1.53	1.04–2.25	0.032*
**Marital status**						
Married	Reference			Reference		
Other	1.23	1.02–1.48	0.029*	1.27	1.04–1.54	0.017*
**Pathological grade**						
I	Reference			Reference		
II	1.41	0.94–2.12	0.101	1.39	0.92–2.10	0.114
III	2.45	1.63–3.69	<0.001*	1.75	1.15–2.64	0.009*
IV	7.25	4.44–11.83	<0.001*	2.20	1.31–3.67	0.003*
**T stage**						
T1/T2	Reference			Reference		
T3/T4	3.82	3.13–4.66	<0.001*	1.36	1.06–1.75	0.015*
**N stage**						
N0	Reference			Reference		
N1	9.32	7.38–11.77	<0.001*	2.21	1.61–3.03	<0.001*
**M stage**						
M0	Reference			Reference		
M1	13.74	10.55–17.91	<0.001*	4.59	3.19–6.59	<0.001*
**Tumor size**						
≤48	Reference			Reference		
49–93	2.25	1.82–2.78	<0.001*	1.42	1.13–1.79	0.003*
≥94	4.80	3.80–6.06	<0.001*	1.78	1.34–2.36	<0.001*
**Chemotherapy**						
Yes	Reference			Reference		
No/Unknown	0.09	0.07–0.13	<0.001*	0.80	0.53–1.21	0.289
**Radiation**						
Yes	Reference			Reference		
No/Unknown	0.10	0.06–0.16	<0.001*	0.95	0.53–1.70	0.865
**Surgery**						
Partial nephrectomy	Reference			Reference		
Radical nephrectomy	3.49	2.85–4.29	<0.001*	2.03	1.60–2.57	<0.001*

HR, hazard ratio; CI, confidence interval;

* , *P* < 0.05.

**Figure 1:**
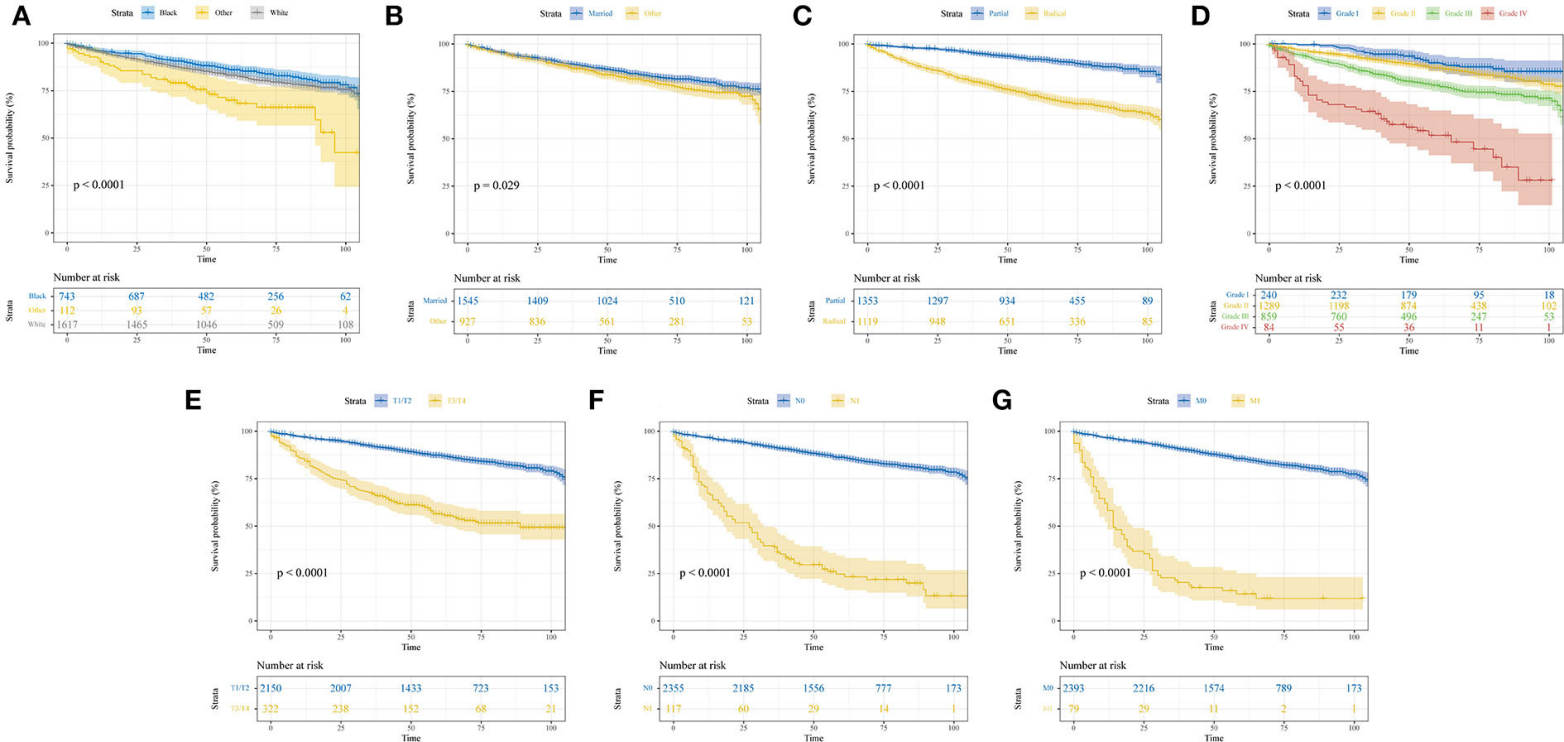
Kaplan–Meier curves show the overall survival of patients with papillary renal cell carcinoma after nephrectomy stratified by different subgroups. **(A)** Race; **(B)** marital status; **(C)** surgery; **(D)** pathological grade; **(E)** T stage; **(F)** N stage; **(G)** M stage.

The median follow-up time was 59 (interquartile range, 42–81) months. A total of 655 patients had end-of-event outcomes, of which 52.2% of deaths were attributable to pRCC. KM survival analysis was conducted to visualize the difference in survival outcomes among different subgroups. Black patients tended to have a longer OS than white patients (*P* < 0.001; Figure 1A). Patients who were married had a better prognosis than other patients, and the difference was statistically significant (*P* = 0.029; Figure 1B). In terms of surgery, patients who underwent partial nephrectomy achieved a significantly longer survival time than patients receiving radical nephrectomy (*P* < 0.001; Figure 1C). As shown in Supplementary Figure 1, higher values of age or tumor size were associated with poor prognosis. Similar to the phenomenon found in age and tumor size, higher pathological grade and TNM stage also led to worse OS (*P* < 0.001; Figures 1D–G).

### Development and validation of the nomogram

**Figure 2:**
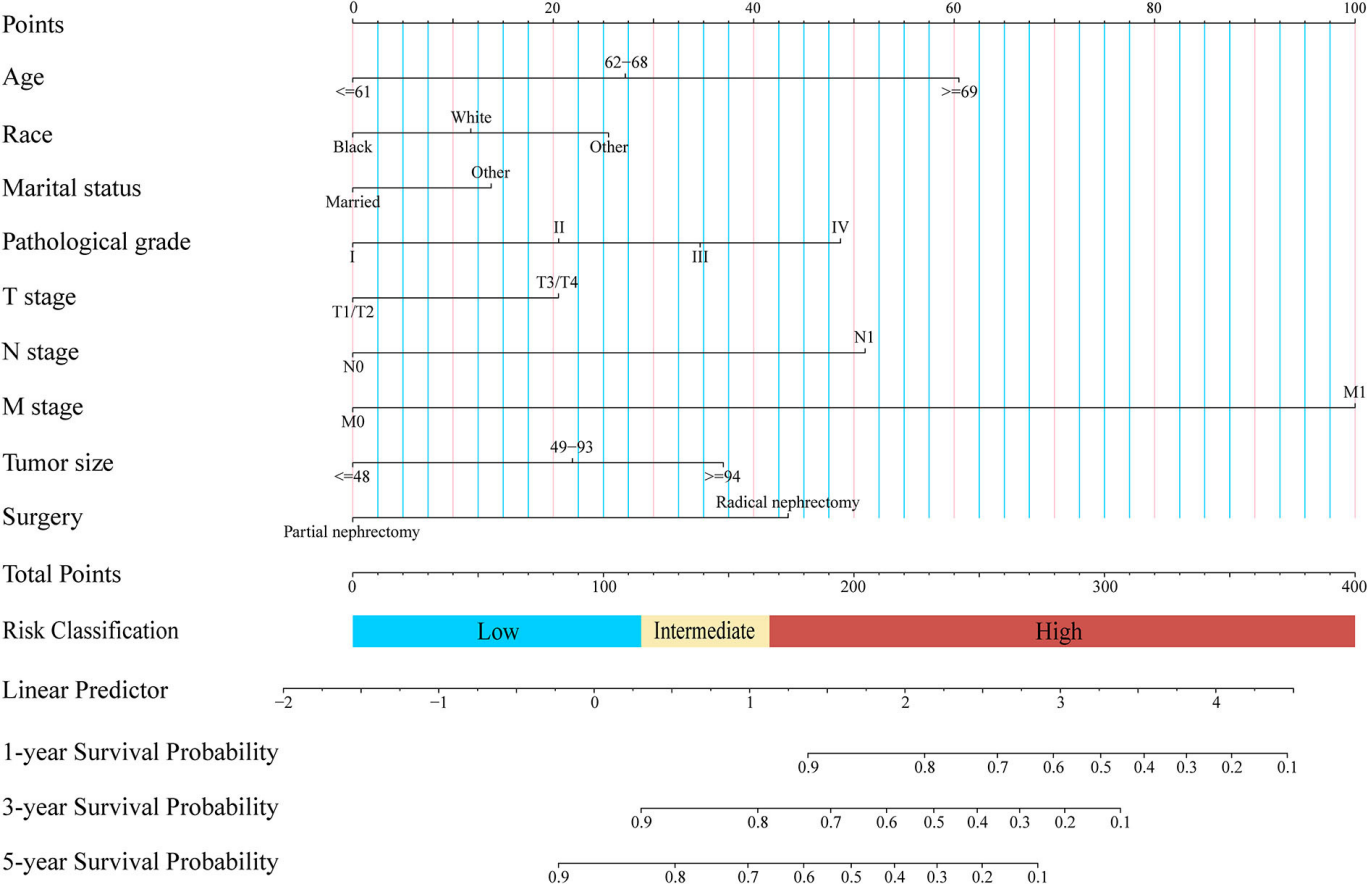
Newly defined nomogram for the overall survival prediction of patients with papillary renal cell carcinoma after nephrectomy.

A clinical prediction nomogram consisting of three demographic variables (age, race, and marital status) and six clinicopathological variables (e.g., pathological grade, TNM stage, tumor size, and surgery) was developed for 1-, 3-, and 5-year OS probabilities (Figure 2). The line length of each variable in the nomogram represented its contribution to OS. The longer the line length, the larger the value of contribution. As shown in the nomogram, the M stage was the strongest prognostic factor, followed by age and N stage. Pathological grade, surgery, and tumor size were moderate contributors, while race, T stage, and marital status made little contribution. Based on the contribution to the survival outcome, each variable had its corresponding point from 0 to 100. The total points could be obtained by adding the points of all variables, and then the individual survival outcome could be predicted through the functional transformation association between the total points and the probability of OS (Table 3).

**Table 3:** Nomogram scoring system.

**Variables**	**Points**	**Variables**	**Points**	**Variables**	**Points**
Age		T stage		Pathological grade	
≤61	0	T1/T2	0	I	0
62–68	27	T3/T4	21	II	21
≥69	60			III	35
Race		N stage		IV	49
Black	0	N0	0	Tumor size	
White	12	N1	51	≤48	0
Other	26			49–93	22
Marital status		M stage		≥94	37
Married	0	M0	0	Surgery	
Other	14	M1	100	Partial nephrectomy	0
				Radical nephrectomy	43
1-year OS probability	Points	3-year OS probability	Points	5-year OS probability	Points
0.9	182	0.9	115	0.9	82
0.8	228	0.8	162	0.8	129
0.7	257	0.6	213	0.6	180
0.5	298	0.5	232	0.5	199
0.4	316	0.4	249	0.4	216
0.3	333	0.3	266	0.3	233
0.2	351	0.2	284	0.2	251
0.1	373	0.1	306	0.1	273

OS, overall survival.

**Figure 3:**
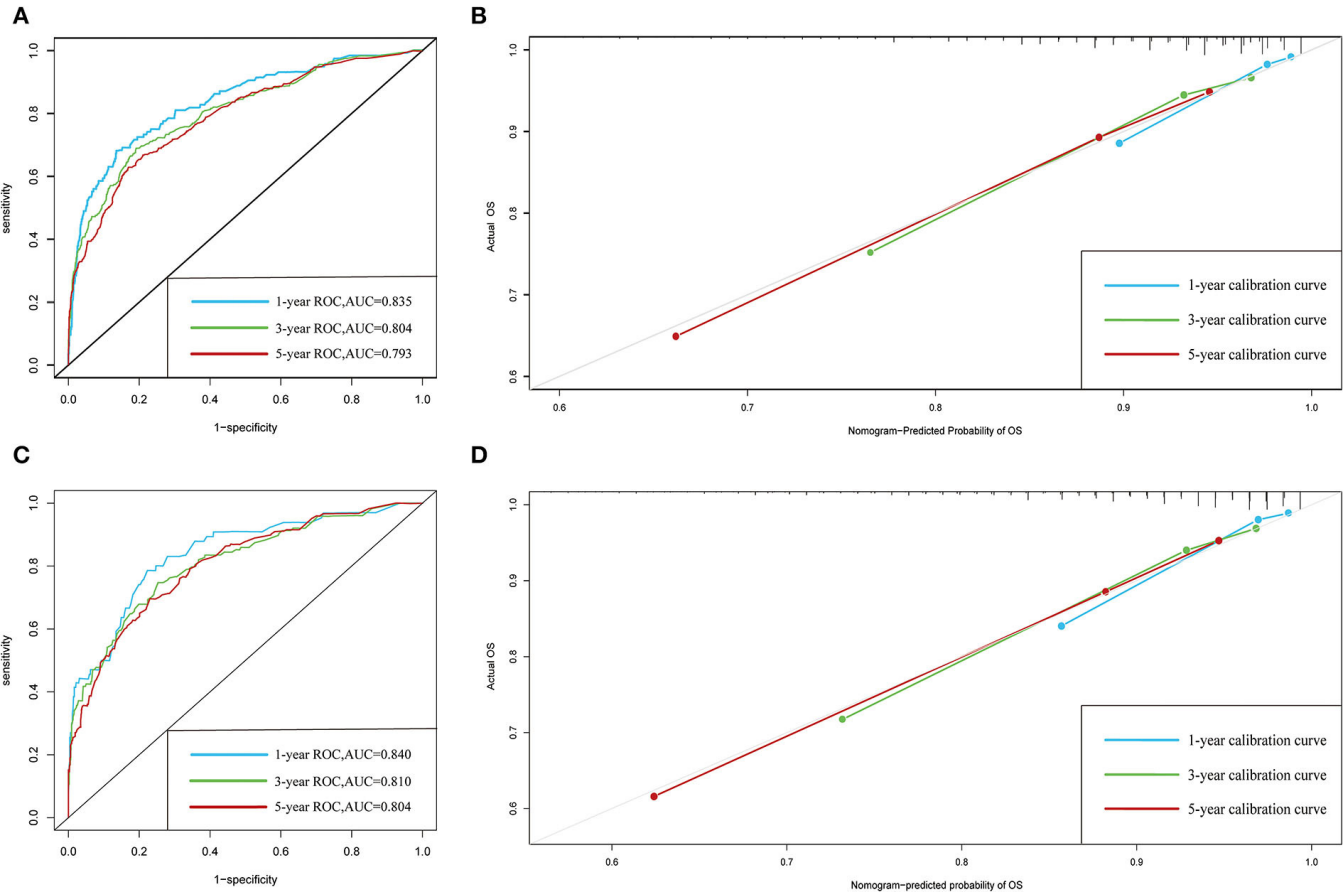
ROC and calibration curves. **(A,B)** ROC and calibration curves of 1-, 3-, and 5-year overall survival in the training cohort. **(C,D)** ROC and calibration curves of 1-, 3-, and 5-year overall survival in the validation cohort.

**Figure 4:**
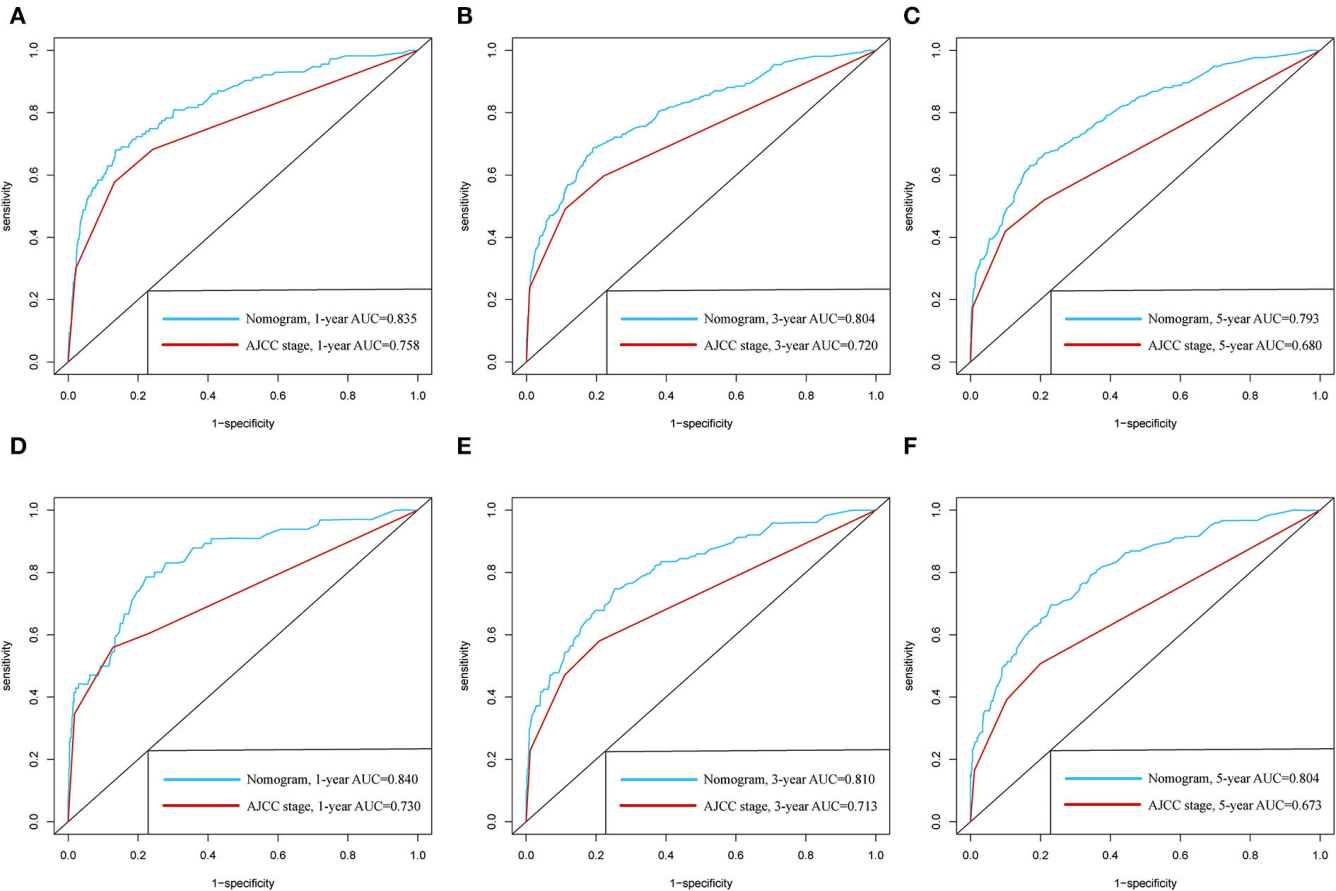
Comparison of the prognostic value of the newly defined nomogram and the traditional AJCC staging by 1-, 3-, and 5-year ROC curves in the training cohort **(A–C)** and the validation cohort **(D–F)**.

**Figure 5:**
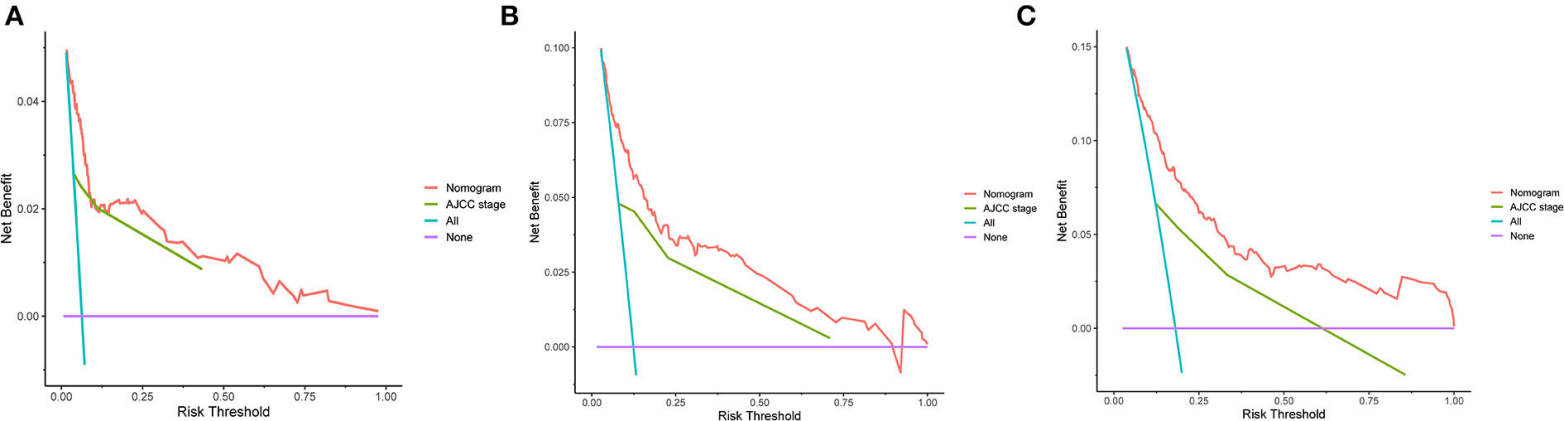
DCA curves. **(A–C)** DCA curves of the nomogram and the traditional AJCC staging for 1-, 3-, and 5-year overall survival.

The C-indexes of the established nomogram were higher than those of the traditional AJCC staging in either the training set (0.777 vs. 0.679) or the validation set (0.787 vs. 0.677). The C-index of the nomogram in the AHMU-pRCC cohort was 0.834. As presented in Figures 3A,C, the values of AUC for 1-, 3-, and 5-year OS showed that the discriminative ability of the nomogram reached acceptable levels in both the training set (1-year: 0.835, 3-year: 0.804, 5-year: 0.793) and validation set (1-year: 0.840, 3-year: 0.810, 5-year: 0.804), which was superior to the traditional AJCC staging (Figure 4). The AUCs for 3- and 5-year OS in the AHMU-pRCC cohort were 0.8 and 0.854, respectively (**Figure 7A**). The calibration plot was also conducted to assess the difference between the nomogram-predicted survival probability and the actual observation, and the results showed that the predicted curves approximatively overlapped with the diagonal line in the three cohorts (Figures 3B,D, 7B), presenting favorable prediction accuracy. DCAs revealed that applying the nomogram to guide clinical practice would provide patients with pRCC with more net benefits than the traditional AJCC staging, especially long-term benefits at 3 and 5 years (Figure 5).

### Risk classification system

**Figure 6:**
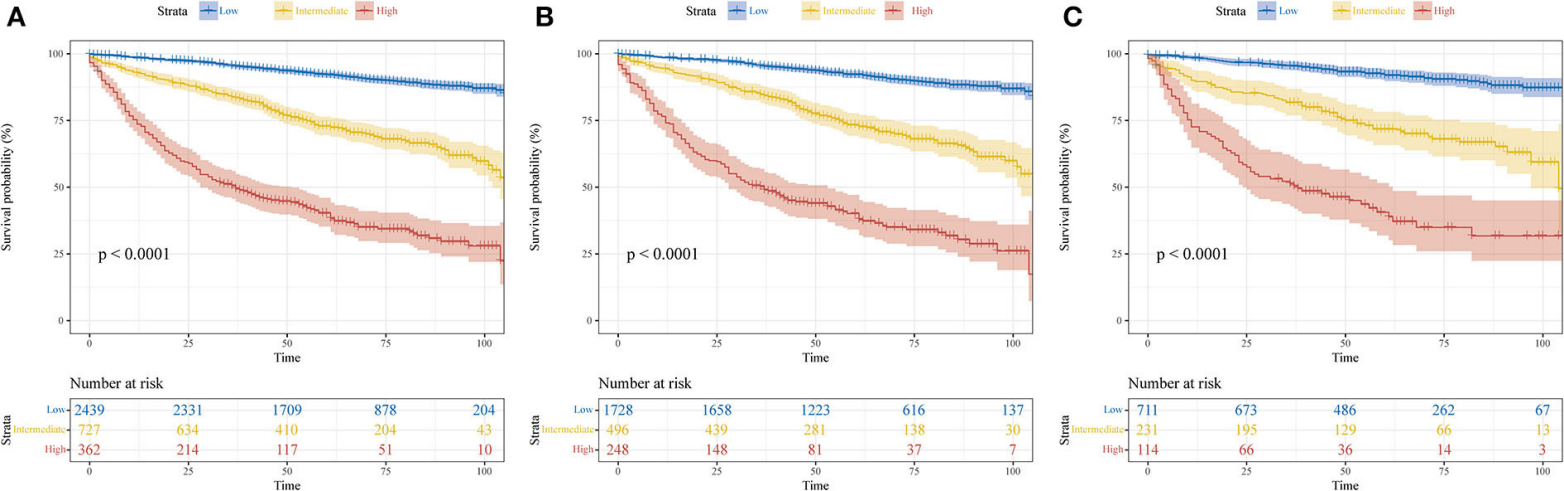
Kaplan–Meier curves show the overall survival of patients with papillary renal cell carcinoma after nephrectomy in low-, intermediate-, and high-risk groups. **(A)** Total cohort; **(B)** training cohort; **(C)** validation cohort.

**Figure 7:**
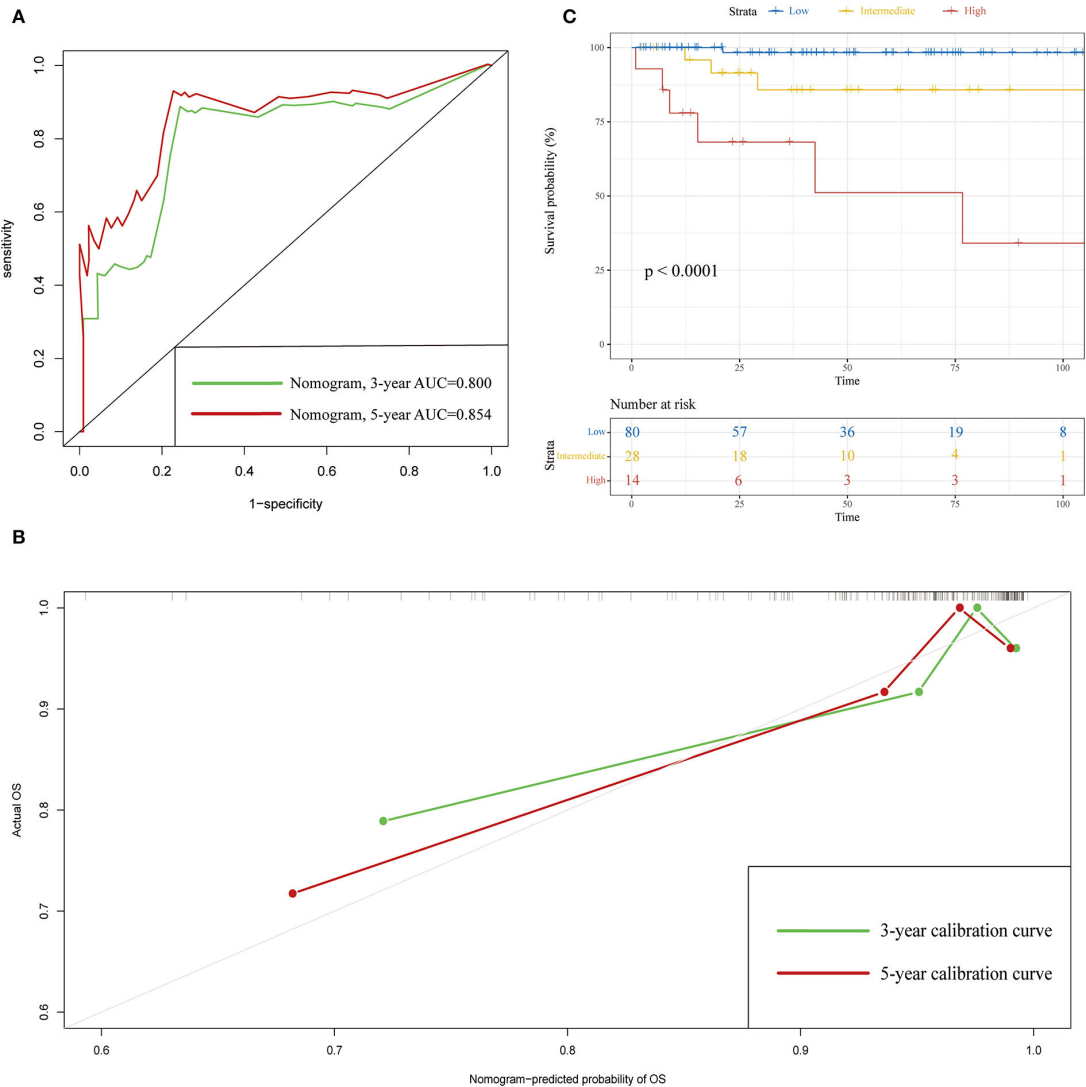
Validation results in the AHMU-pRCC cohort. **(A)** ROC curves of 3- and 5-year overall survival. **(B)** Calibration curves of 3- and 5-year overall survival. **(C)** Kaplan–Meier curves show the overall survival of patients with papillary renal cell carcinoma after nephrectomy in the low-, intermediate-, and high-risk groups.

To further optimize the clinical application of the nomogram, a risk classification system produced by X-tile software was built based on the total points of each patient in the training cohort (Supplementary Figure 3). According to the novel risk classification system, patients with pRCC in the training cohort were divided into three risk levels: low-risk group (1,728/2,472, 70%; <115 points), intermediate-risk group (496/2,472, 20%; 115–168 points), and high-risk group (248/2,472, 10%; >168 points). We also utilized the risk classification system to classify patients in the validation cohort and the total cohort. In the total cohort, as the level of risk increased, the decline in survival probability was significant (Figure 6A). KM plots for overall survival outcomes showed that the OS of the low-, intermediate-, and high-risk groups in the training cohort was significantly discriminated (Figure 6B), and the discriminative ability was confirmed in the validation cohort and AHMU-pRCC cohort (Figures 6C, 7C).

## Discussion

The incidence of kidney cancer has gradually increased during the past few decades, and thus, pRCC, as the second most common kidney cancer subtype, poses an increasing threat to human health (26). Nephrectomy is a common surgical procedure for the treatment of kidney cancer, mainly including partial nephrectomy and radical nephrectomy. With the rapid development of laparoscopic and artificial intelligence technology, minimally invasive techniques such as laparoscopic nephrectomy and robot-assisted nephrectomy have replaced open nephrectomy as the main surgical procedures. There is no significant difference in the therapeutic effect between open and minimally invasive nephrectomy, but minimally invasive nephrectomy is superior to open nephrectomy in terms of intraoperative bleeding, length of hospital stay, analgesic requirements, and enhanced postoperative recovery (5, 27–30). Although the survival of patients with pRCC has been significantly improved by the innovations of minimally invasive surgery, accurate assessment of postoperative prognosis remains a complex task for clinicians. Identifying the risk factors affecting the prognosis of pRCC after nephrectomy is of great significance for facilitating individualized and precise treatment.

The AJCC staging is currently the most commonly used clinical tool for assessing the prognosis of patients with pRCC. However, the limitations of this traditional clinical staging system are evident because the system incorporates fewer clinical parameters to measure the overall situation of patients with pRCC (31). It is true that the survival outcome is reflected not only by the TNM stage in the traditional AJCC staging but also by other prognostic factors, including age, sex, race, marital status, pathological grade, surgery, and adjunctive therapy. A nomogram based on AJCC staging in combination with other important clinical indicators has been widely applied as a convenient and effective tool to quantitatively predict survival time, and its accuracy and reliability have been validated in multiple cancers (32–35). Our previous studies also identified novel prognosis-related biomarkers and established nomograms to predict the recurrence-free survival of patients with RCC (36–38). In the present study, we attempted to construct a prognostic nomogram for predicting the OS of patients with pRCC after nephrectomy for the first time by using readily available clinical data.

Multivariate Cox analysis in the training cohort showed that three demographic variables (age, race, and marital status) and six clinicopathological variables (e.g., pathological grade, TNM stage, tumor size, and surgery) were significant OS-related prognostic factors, all of which being incorporated into the construction of the nomogram. High C-indexes and AUC values of the nomogram at 1, 3, and 5 years were identified in the three cohorts, indicating that the nomogram had satisfactory accuracy and discriminative ability. However, high accuracy and discriminative ability have no association with the performance of clinical utility. Thus, DCA was performed, and the results confirmed that the nomogram obtained more net benefits than the AJCC staging, especially long-term benefits at 3 and 5 years. Furthermore, a risk classification system based on the nomogram was introduced, and the discrimination was confirmed in the validation cohort and AHMU-pRCC cohort. With the help of this system, clinicians can risk-stratify patients with pRCC accurately and make appropriate interventions to improve the prognosis of patients. We believe that the following reasons may explain why the nomogram has higher clinical practicability. First, all the included patients with pRCC received partial nephrectomy or radical nephrectomy, so the nomogram derived from these patients was more representative. Second, the involvement of age, race, marital status, pathological grade, tumor size, and surgery made the nomogram more accurate in reflecting the overall situation of patients and thus effectively predicting OS. As presented in the nomogram, age, pathological grade, and surgery were the second, fourth, and fifth largest contributors, further illustrating that other demographic and clinicopathological variables in addition to TNM stage were also of significant importance in predicting prognosis. Third, we used risk points to show the weight of different prognostic factors and presented them in a graphic model, which is convenient for clinicians to apply in clinical practice.

Age has been proven to be a crucial factor for prognosis in many other cancers (39–41) since elderly patients often found to have more comorbidities, such as cardiovascular disease, pulmonary dysfunction, and metabolic disorders, all of which can lead to the development of disease. Meanwhile, the elderly are often diagnosed at an advanced stage due to the lack of effective monitoring. Consistent with the aforementioned findings, age was identified as the second largest contributor to prognosis, and OS decreased significantly with increasing age. Therefore, enough attention should be given to elderly patients with pRCC after nephrectomy in clinical practice. It has been reported that African Americans with RCC have low survival rates because of limited financial resources and poor accessibility to medical care (42). In contrast to these results, we did not find that the black race was related to a low OS. Conversely, black patients have more favorable survival outcomes than other races, suggesting that the previous conclusion may not be applicable for pRCC. A large population-based study performed by Huang et al. showed that non-Hispanic blacks with metastatic pRCC had a higher survival rate than non-Hispanic whites with metastatic pRCC, further proving our conclusion (14). In addition to age and race, marital status is another important demographic indicator that affects the natural history of many cancers (43, 44). In the present study, we investigated the effect of marital status on the OS of patients with pRCC who underwent nephrectomy and assessed the magnitude of this effect. We observed that married patients with pRCC presented with improved survival. A proposed mechanism for explaining this phenomenon was that married patients with pRCC received more financial and emotional support than unmarried patients (45).

Higher clinical stages are related to poor prognosis, and distant metastasis is the strongest prognostic factor among all included factors. However, tumor size in patients with RCC was associated with metastasis and malignant potential (46, 47), which could explain why patients with large tumor sizes, especially those with tumors larger than 94 mm, had unfavorable survival outcomes. With regard to treatment modality, the oncologic outcomes of patients treated with radical nephrectomy were more unfavorable than those treated with partial nephrectomy. We consider this may be because patients who receive radical nephrectomy usually have tumors that are located in complex sites or invade the surrounding organs. For these patients, there is a high possibility of distant metastasis even after surgery (48), so early diagnosis and treatment are of great significance to improve the prognosis of pRCC. Notably, neither radiation nor chemotherapy is effective in treating RCC (5). Although radiation and chemotherapy were significant in univariate Cox analysis, subsequent multivariate Cox analyses failed to identify them as independent risk factors for pRCC prognosis. Recent studies have shown that tyrosine kinase inhibitors for the VEGF signaling pathway, such as sunitinib and cabozantinib, have promising clinical applications in advanced non-clear-cell RCC (49–51). Due to the lack of data on targeted therapy in the SEER database, we could not conduct in-depth research.

Klatte et al. first developed and validated a prognostic nomogram for patients with pRCC after nephrectomy, but the primary endpoint of their study was disease-specific survival, and not OS (52). Moreover, the nomogram may not be generally applicable in that it lacks a large sample size and an assessment of clinical net benefits. Su et al. developed the first competing risk survival nomogram to predict cancer-specific mortality in patients with pRCC after surgery and found that elderly patients had few long-term benefits from treatment and were associated with poor prognosis (53), which was in line with our results. Recently, Yan et al. reported two survival nomograms to predict the 3- and 5-year OS and cancer-specific survival probabilities for patients with pRCC (54). Of note, the patients included in their study were not all treated with nephrectomy, and thus, the nomogram based on these patients was not representative compared to our model. Meanwhile, the nomogram is limited because the C-index, ROC, or DCA was not used to determine whether the model had more advantages over the AJCC staging. In addition, other important indicators, including race, marital status, and pathological grade, were not included, which could affect the accuracy of the nomogram. Therefore, it is not surprising that the C-indexes and AUCs for 3- and 5-year OS in their study were lower than those of our nomogram.

Although the present study developed the first nomogram regarding the prediction of OS of patients with pRCC after nephrectomy, the limitations should also be noted. Primarily, the 1-year results were not externally validated due to very few deaths within the 1-year follow-up. In addition, external validation in the study was based on small sample data. Multicenter cohorts containing a larger sample size are warranted to further confirm the performance of the nomogram. Furthermore, the histological subtypes of pRCC were not considered, which might impact the accuracy of the nomogram. Finally, other potential prognostic factors, including laboratory data, surgical margin status, disease recurrence, targeted therapy, and immunotherapy, were not included in the nomogram, all of which should be evaluated in future studies.

## Conclusion

Age, race, marital status, TNM stage, tumor size, and surgery were significantly associated with the OS of patients with pRCC after nephrectomy. Based on these easily available factors in clinical practice, a predictive nomogram with the corresponding risk classification system was developed, which had high accuracy and practicability. With the guidance of the nomogram, more optimized decision-making will be conducted to improve the prognosis of postoperative patients with pRCC.

## Data availability statement

The original contributions presented in the study are included in the article/Supplementary material, further inquiries can be directed to the corresponding authors.

## Ethics statement

The study was approved by the Ethics Committee of The First Affiliated Hospital of Anhui Medical University (PJ-2022-11-20). Written informed consent for participation was not required for this study in accordance with the national legislation and the institutional requirements.

## Author contributions

YH, SX, and QQ designed this study and wrote this manuscript. SX, XW, JM, and QL collected the data. YH, QQ, JZ, and ZH integrated and analyzed the data. CL and XF edited and revised the manuscript. All authors approved the final manuscript.

## Funding

This study received funding from the National Natural Science Foundation of China (82170787, 81870519, and 81630019); Scientific Research Foundation of the Institute for Translational Medicine of Anhui Province (2017ZHYX02); Supporting Project for Distinguished Young Scholars of Anhui Colleges (gxyqZD2019018); and Youth Talent Science and Technology Project of Changzhou Health Commission (QN202109).

## Conflict of interest

The authors declare that the research was conducted in the absence of any commercial or financial relationships that could be construed as a potential conflict of interest.

## Publisher's note

All claims expressed in this article are solely those of the authors and do not necessarily represent those of their affiliated organizations, or those of the publisher, the editors and the reviewers. Any product that may be evaluated in this article, or claim that may be made by its manufacturer, is not guaranteed or endorsed by the publisher.

## Supplementary material

Supplementary Figure 1 X-tile analysis. **(A–D)** The best cut-off values of age and tumor size were determined by X-tile software. **(A,D)** X-tile plots of age and tumor size in the training cohort. **(B,E)** Histograms and **(C,F)** Kaplan–Meier plots were generated according to the cut-off values.

Supplementary Figure 2 Forest plot showing the results of multivariate Cox regression analysis in different subgroups.

Supplementary Figure 3 X-tile analysis. **(A,B)** The best cut-off value of the total points determined by X-tile software. **(A)** X-tile plot of the total points in the training cohort. **(B)** A histogram was generated according to the cut-off value.

## References

[1] Siegel RL Miller KD Fuchs HE Jemal A. Cancer statistics, 2022. CA Cancer J Clin. (2022) 72:7–33. 10.3322/caac.21708 35020204

[2] Steffens S Janssen M Roos FC Becker F Schumacher S Seidel C. Incidence and long-term prognosis of papillary compared to clear cell renal cell carcinoma–a multicentre study. Eur J Cancer. (2012) 48:2347–52. 10.1016/j.ejca.2012.05.002 22698386

[3] Sung H Ferlay J Siegel RL Laversanne M Soerjomataram I Jemal A. Global cancer statistics 2020: globocan estimates of incidence and mortality worldwide for 36 cancers in 185 countries. CA Cancer J Clin. (2021) 71:209–49. 10.3322/caac.21660 33538338

[4] Hsieh JJ Purdue MP Signoretti S Swanton C Albiges L Schmidinger M. Renal cell carcinoma. Nat Rev Dis Primers. (2017) 3:17009. 10.1038/nrdp.2017.9 28276433 PMC5936048

[5] Ljungberg B Albiges L Abu-Ghanem Y Bensalah K Dabestani S Fernández-Pello S. European association of urology guidelines on renal cell carcinoma: the 2019 update. Eur Urol. (2019) 75:799–810. 10.1016/j.eururo.2019.02.011 30803729

[6] Siegel RL Miller KD Jemal A. Cancer statistics, 2018. CA Cancer J Clin. (2018) 68:7–30. 10.3322/caac.21442 29313949

[7] Capitanio U Montorsi F. Renal cancer. Lancet. (2016) 387:894–906. 10.1016/s0140-6736(15)00046-x 26318520

[8] Choueiri TK Motzer RJ. Systemic therapy for metastatic renal-cell carcinoma. N Engl J Med. (2017) 376:354–66. 10.1056/NEJMra1601333 28121507

[9] Akhtar M Al-Bozom IA Al Hussain T. Papillary renal cell carcinoma (PRCC): an update. Adv Anat Pathol. (2019) 26:124–32. 10.1097/pap.0000000000000220 30507616

[10] Cairns P. Renal cell carcinoma. Cancer Biomark. (2010) 9:461–73. 10.3233/cbm-2011-0176 22112490 PMC3308682

[11] Kaldany A Paulucci DJ Kannappan M Beksac AT Anastos H Okhawere K. Clinicopathological and survival analysis of clinically advanced papillary and chromophobe renal cell carcinoma. Urol Oncol. (2019) 37:727–34. 10.1016/j.urolonc.2019.05.008 31174958

[12] Keegan KA Schupp CW Chamie K Hellenthal NJ Evans CP Koppie TM. Histopathology of surgically treated renal cell carcinoma: survival differences by subtype and stage. J Urol. (2012) 188:391–7. 10.1016/j.juro.2012.04.006 22698625 PMC3714400

[13] Wagener N Edelmann D Benner A Zigeuner R Borgmann H Wolff I. Outcome of papillary versus clear cell renal cell carcinoma varies significantly in non-metastatic disease. PLoS ONE. (2017) 12:e0184173. 10.1371/journal.pone.0184173 28934212 PMC5608215

[14] Huang J Huang D Yan J Chen T Gao Y Xu D. Comprehensive subgroup analyses of survival outcomes between clear cell renal cell adenocarcinoma and papillary renal cell adenocarcinoma. Cancer Med. (2020) 9:9409–18. 10.1002/cam4.3563 33141518 PMC7774724

[15] Rosiello G Palumbo C Knipper S Pecoraro A Luzzago S St-Hilaire PA. Comparison of survival outcomes in patients with metastatic papillary vs. clear-cell renal cell carcinoma: a propensity-score analysis. World J Urol. (2021) 39:461–72. 10.1007/s00345-020-03187-y 32253579

[16] Attalla K Voss MH Hakimi AA. Prognostic models in papillary renal cell carcinoma. Ann Transl Med. (2020) 8:1334. 10.21037/atm-20-3750 33313079 PMC7723602

[17] Wu WT Li YJ Feng AZ Li L Huang T Xu AD. Data mining in clinical big data: the frequently used databases, steps, and methodological models. Mil Med Res. (2021) 8:44. 10.1186/s40779-021-00338-z 34380547 PMC8356424

[18] Yang J Li Y Liu Q Li L Feng A Wang T. Brief introduction of medical database and data mining technology in big data era. J Evid Based Med. (2020) 13:57–69. 10.1111/jebm.12373 32086994 PMC7065247

[19] Camp RL Dolled-Filhart M Rimm DL. X-tile: a new bio-informatics tool for biomarker assessment and outcome-based cut-point optimization. Clin Cancer Res. (2004) 10:7252–9. 10.1158/1078-0432.Ccr-04-0713 15534099

[20] Christensen E. Multivariate survival analysis using cox's regression model. Hepatology. (1987) 7:1346–58. 10.1002/hep.1840070628 3679094

[21] Lin DY. Cox regression analysis of multivariate failure time data: the marginal approach. Stat Med. (1994) 13:2233–47. 10.1002/sim.4780132105 7846422

[22] Alba AC Agoritsas T Walsh M Hanna S Iorio A Devereaux PJ. Discrimination and calibration of clinical prediction models: users' guides to the medical literature. JAMA. (2017) 318:1377–84. 10.1001/jama.2017.12126 29049590

[23] Iasonos A Schrag D Raj GV Panageas KS. How to build and interpret a nomogram for cancer prognosis. J Clin Oncol. (2008) 26:1364–70. 10.1200/jco.2007.12.9791 18323559

[24] Vickers AJ Elkin EB. Decision curve analysis: a novel method for evaluating prediction models. Med Decis Making. (2006) 26:565–74. 10.1177/0272989x06295361 17099194 PMC2577036

[25] Wang Y Li J Xia Y Gong R Wang K Yan Z. Prognostic nomogram for intrahepatic cholangiocarcinoma after partial hepatectomy. J Clin Oncol. (2013) 31:1188–95. 10.1200/jco.2012.41.5984 23358969

[26] Palumbo C Pecoraro A Knipper S Rosiello G Luzzago S Deuker M. Contemporary age-adjusted incidence and mortality rates of renal cell carcinoma: analysis according to gender, race, stage, grade, and histology. Eur Urol Focus. (2021) 7:644–52. 10.1016/j.euf.2020.05.003 32456993

[27] Makhoul B De La Taille A Vordos D Salomon L Sebe P Audet JF. Laparoscopic radical nephrectomy for t1 renal cancer: the gold standard? A comparison of laparoscopic vs open nephrectomy. BJU Int. (2004) 93:67–70. 10.1111/j.1464-410x.2004.04558.x 14678371

[28] Sprenkle PC Power N Ghoneim T Touijer KA Dalbagni G Russo P. Comparison of open and minimally invasive partial nephrectomy for renal tumors 4-7 centimeters. Eur Urol. (2012) 61:593–9. 10.1016/j.eururo.2011.11.040 22154728 PMC6693652

[29] Laird A Choy KC Delaney H Cutress ML O'Connor KM Tolley DA. Matched pair analysis of laparoscopic versus open radical nephrectomy for the treatment of T3 renal cell carcinoma. World J Urol. (2015) 33:25–32. 10.1007/s00345-014-1280-y 24647880

[30] Patel P Nayak JG Liu Z Saarela O Jewett M Rendon R. A multicentered, propensity matched analysis comparing laparoscopic and open surgery for Pt3a renal cell carcinoma. J Endourol. (2017) 31:645–50. 10.1089/end.2016.0787 28381117

[31] Ficarra V Galfano A Mancini M Martignoni G Artibani W. TNM staging system for renal-cell carcinoma: current status and future perspectives. Lancet Oncol. (2007) 8:554–8. 10.1016/s1470-2045(07)70173-0 17540307

[32] Groot Koerkamp B Wiggers JK Gonen M Doussot A Allen PJ Besselink MGH. Survival after resection of perihilar cholangiocarcinoma-development and external validation of a prognostic nomogram. Ann Oncol. (2015) 26:1930–5. 10.1093/annonc/mdv279 26133967 PMC4754626

[33] Li X Xu H Yan L Gao J Zhu L. A novel clinical nomogram for predicting cancer-specific survival in adult patients after primary surgery for epithelial ovarian cancer: a real-world analysis based on the surveillance, epidemiology, and end results database and external validation in a tertiary center. Front Oncol. (2021) 11:670644. 10.3389/fonc.2021.670644 33959514 PMC8093627

[34] Han DS Suh YS Kong SH Lee HJ Choi Y Aikou S. Nomogram predicting long-term survival after D2 gastrectomy for gastric cancer. J Clin Oncol. (2012) 30:3834–40. 10.1200/jco.2012.41.8343 23008291

[35] Liang W Zhang L Jiang G Wang Q Liu L Liu D. Development and validation of a nomogram for predicting survival in patients with resected non-small-cell lung cancer. J Clin Oncol. (2015) 33:861–9. 10.1200/jco.2014.56.6661 25624438

[36] Feng X Zhang M Meng J Wang Y Liu Y Liang C. Correlating transcriptional networks to papillary renal cell carcinoma survival: a large-scale coexpression analysis and clinical validation. Oncol Res. (2020) 28:285–97. 10.3727/096504020x15791676105394 31948514 PMC7851515

[37] Guan Y Wang B Zhang T Gao S Cao Z Zhang M. Integrated analysis revealed the microrna-based prognostic predicting signature for papillary renal cell carcinoma. DNA Cell Biol. (2021) 40:532–42. 10.1089/dna.2019.5306 33625263

[38] Bian Z Meng J Niu Q Jin X Wang J Feng X. Prognostic role of prothrombin time activity, prothrombin time, albumin/globulin ratio, platelets, sex, and fibrinogen in predicting recurrence-free survival time of renal cancer. Cancer Manag Res. (2020) 12:8481–90. 10.2147/cmar.S264856 32982441 PMC7505717

[39] Eguchi T Bains S Lee MC Tan KS Hristov B Buitrago DH. Impact of increasing age on cause-specific mortality and morbidity in patients with stage I non-small-cell lung cancer: a competing risks analysis. J Clin Oncol. (2017) 35:281–90. 10.1200/jco.2016.69.0834 28095268 PMC5456376

[40] Yamano T Yamauchi S Kimura K Babaya A Hamanaka M Kobayashi M. Influence of age and comorbidity on prognosis and application of adjuvant chemotherapy in elderly Japanese patients with colorectal cancer: a retrospective multicentre study. Eur J Cancer. (2017) 81:90–101. 10.1016/j.ejca.2017.05.024 28622612

[41] Pettersson A Robinson D Garmo H Holmberg L Stattin P. Age at diagnosis and prostate cancer treatment and prognosis: a population-based cohort study. Ann Oncol. (2018) 29:377–85. 10.1093/annonc/mdx742 29161337

[42] Anastos H Martini A Waingankar N Paulucci DJ Beksac AT Daza J. Black race may be associated with worse overall survival in renal cell carcinoma patients. Urol Oncol. (2020) 38:938.e9–17. 10.1016/j.urolonc.2020.08.034 32950398

[43] Wu C Chen P Qian JJ Jin SJ Yao J Wang XD. Effect of marital status on the survival of patients with hepatocellular carcinoma treated with surgical resection: an analysis of 13,408 patients in the surveillance, epidemiology, and end results (Seer) database. Oncotarget. (2016) 7:79442–52. 10.18632/oncotarget.12722 27769053 PMC5346726

[44] Tao L Pan X Zhang L Wang J Zhang Z Zhang L. Marital status and prognostic nomogram for bladder cancer with distant metastasis: a seer-based study. Front Oncol. (2020) 10:586458. 10.3389/fonc.2020.586458 33194738 PMC7654226

[45] Aizer AA Chen MH McCarthy EP Mendu ML Koo S Wilhite TJ. Marital status and survival in patients with cancer. J Clin Oncol. (2013) 31:3869–76. 10.1200/jco.2013.49.6489 24062405 PMC4878087

[46] Thompson RH Hill JR Babayev Y Cronin A Kaag M Kundu S. Metastatic renal cell carcinoma risk according to tumor size. J Urol. (2009) 182:41–5. 10.1016/j.juro.2009.02.128 19450840 PMC2735023

[47] Thompson RH Kurta JM Kaag M Tickoo SK Kundu S Katz D. Tumor size is associated with malignant potential in renal cell carcinoma cases. J Urol. (2009) 181:2033–6. 10.1016/j.juro.2009.01.027 19286217 PMC2734327

[48] Dabestani S Thorstenson A Lindblad P Harmenberg U Ljungberg B Lundstam S. Renal cell carcinoma recurrences and metastases in primary non-metastatic patients: a population-based study. World J Urol. (2016) 34:1081–6. 10.1007/s00345-016-1773-y 26847337

[49] Martínez Chanzá N Xie W Asim Bilen M Dzimitrowicz H Burkart J Geynisman DM. Cabozantinib in advanced non-clear-cell renal cell carcinoma: a multicentre, retrospective, cohort study. Lancet Oncol. (2019) 20:581–90. 10.1016/s1470-2045(18)30907-0 30827746 PMC6849381

[50] Choueiri TK Plantade A Elson P Negrier S Ravaud A Oudard S. Efficacy of sunitinib and sorafenib in metastatic papillary and chromophobe renal cell carcinoma. J Clin Oncol. (2008) 26:127–31. 10.1200/jco.2007.13.3223 18165647

[51] Tannir NM Plimack E Ng C Tamboli P Bekele NB Xiao L. A phase 2 trial of sunitinib in patients with advanced non-clear cell renal cell carcinoma. Eur Urol. (2012) 62:1013–9. 10.1016/j.eururo.2012.06.043 22771265 PMC3882163

[52] Klatte T Remzi M Zigeuner RE Mannweiler S Said JW Kabbinavar FF. Development and external validation of a nomogram predicting disease specific survival after nephrectomy for papillary renal cell carcinoma. J Urol. (2010) 184:53–8. 10.1016/j.juro.2010.03.026 20478577

[53] Su X Hou NN Yang LJ Li PX Yang XJ Hou GD. The first competing risk survival nomogram in patients with papillary renal cell carcinoma. Sci Rep. (2021) 11:11835. 10.1038/s41598-021-91217-z 34088935 PMC8178392

[54] Yan H Wei X Wu A Sha Y Li X Qi F. Nomograms for predicting overall and cancer-specific survival in patients with papillary renal cell carcinoma: a population-based study using seer database. Transl Androl Urol. (2020) 9:1146–58. 10.21037/tau-19-807 32676398 PMC7354311

