# Supplementary figures and images for: A novel nomogram and risk classification system predicting the overall survival of patients with papillary renal cell carcinoma after nephrectomy: A population-based study
Source: Front Public Health. 2022 Oct 5;10:989566. doi: 10.3389/fpubh.2022.989566 (PMC9581403; doi:10.3389/fpubh.2022.989566)

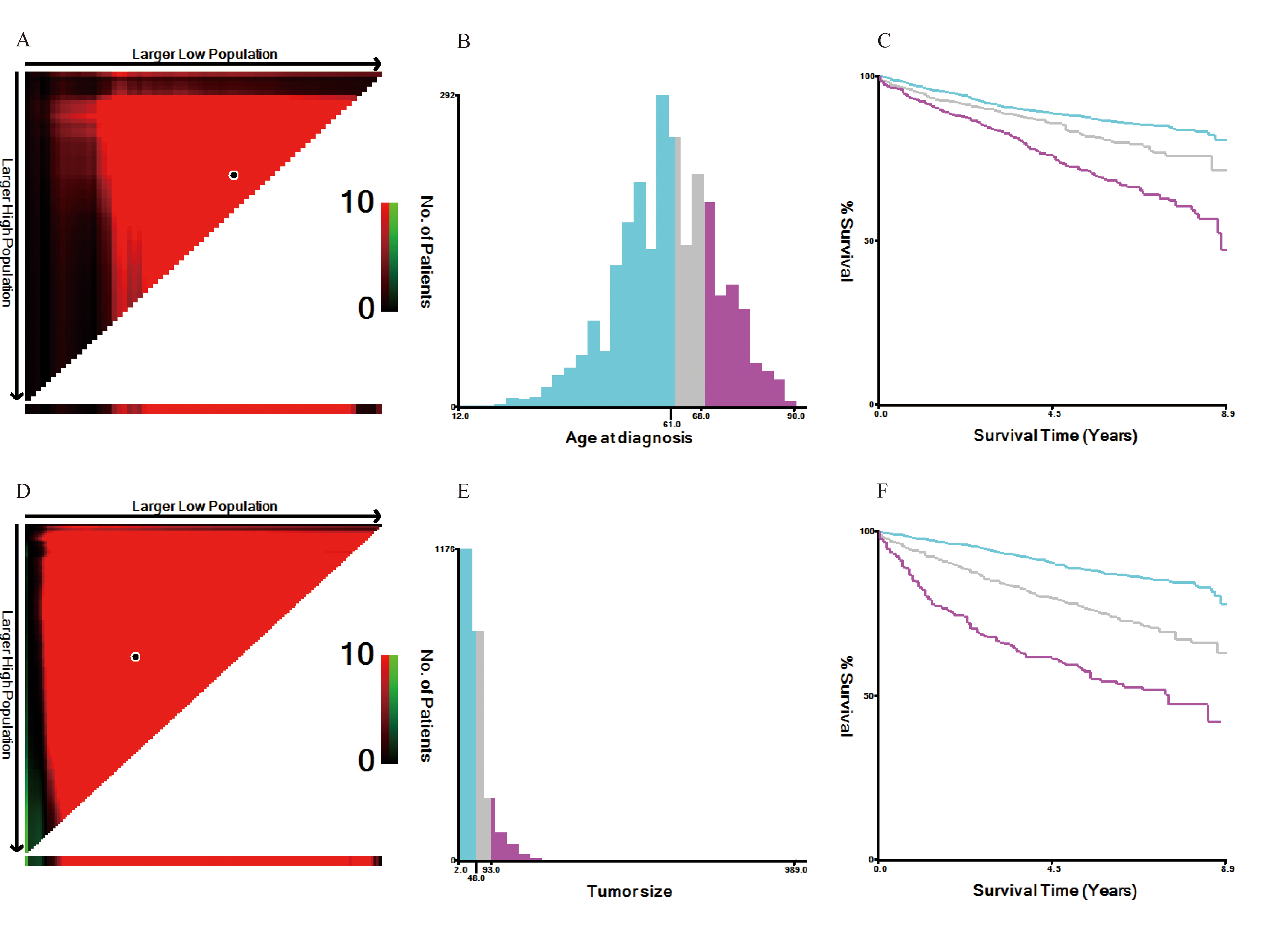

Supplement: Supplementary Figure 1 — X-tile analysis. (A–D) The best cut-off values of age and tumor size were determined by X-tile software. (A,D) X-tile plots of age and tumor size in the training cohort. (B,E) Histograms and (C,F) Kaplan–Meier plots were generated according to the cut-off values. [file Image_1.TIF]

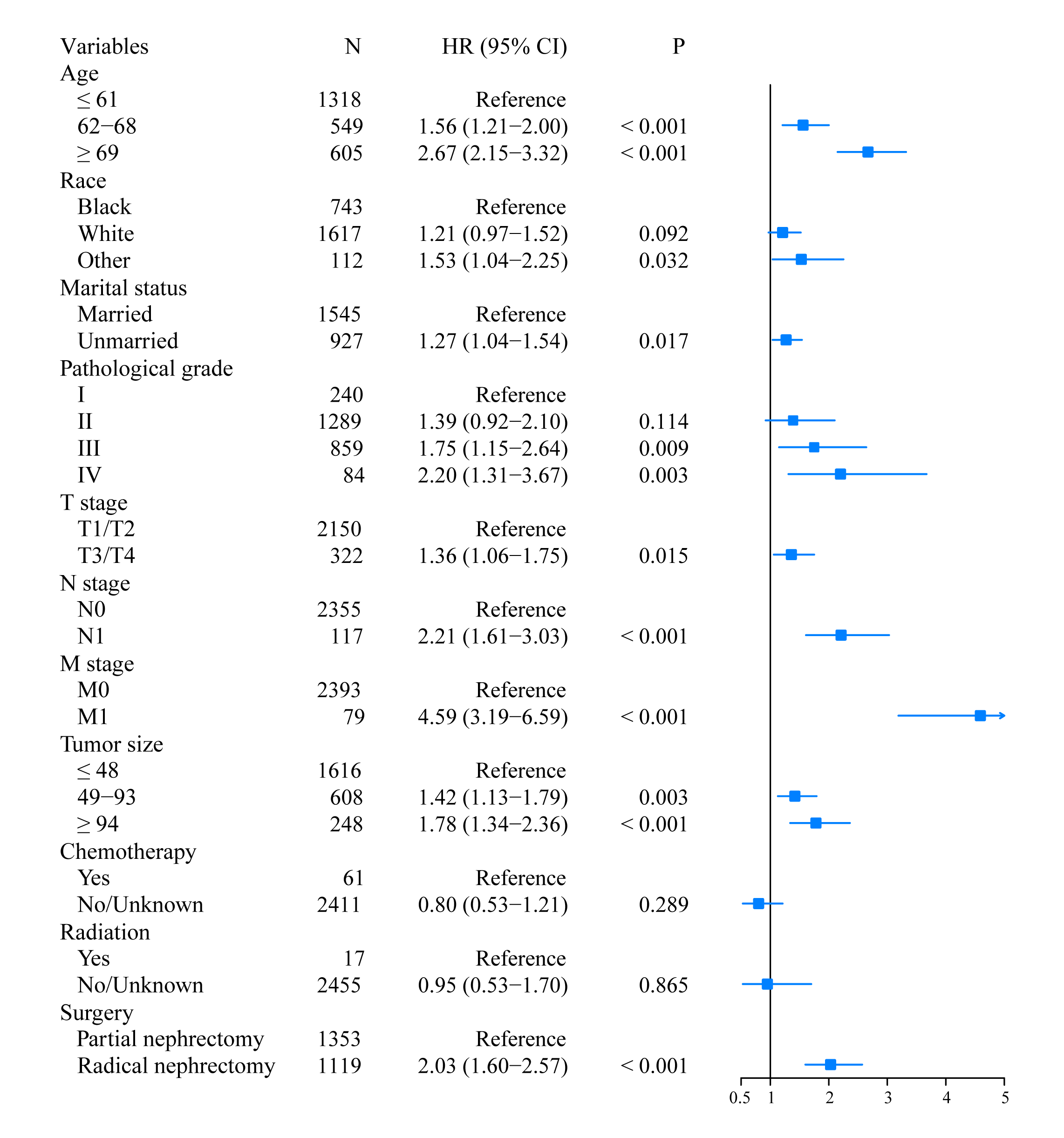

Supplement: Supplementary Figure 2 — Forest plot showing the results of multivariate Cox regression analysis in different subgroups. [file Image_2.TIF]

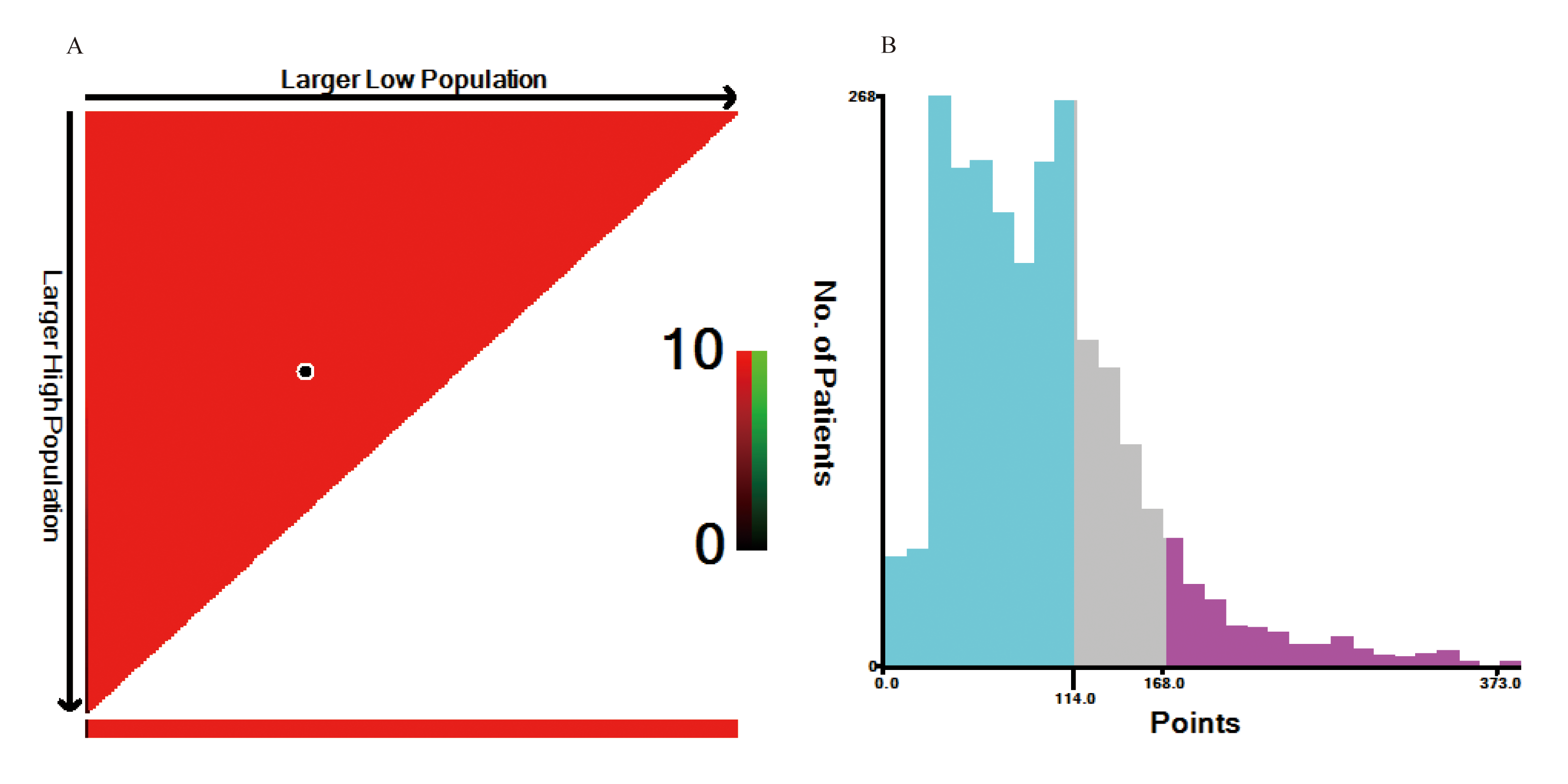

Supplement: Supplementary Figure 3 — X-tile analysis. (A,B) The best cut-off value of the total points determined by X-tile software. (A) X-tile plot of the total points in the training cohort. (B) A histogram was generated according to the cut-off value. [file Image_3.TIF]
